# Comparison of Soil Respiration in Typical Conventional and New Alternative Cereal Cropping Systems on the North China Plain

**DOI:** 10.1371/journal.pone.0080887

**Published:** 2013-11-20

**Authors:** Bing Gao, Xiaotang Ju, Fang Su, Fengbin Gao, Qingsen Cao, Oene Oenema, Peter Christie, Xinping Chen, Fusuo Zhang

**Affiliations:** 1 College of Resources and Environmental Sciences, China Agricultural University, Beijing, China; 2 Wageningen University and Research Center, Alterra, Wageningen, The Netherlands; University of Maryland, United States of America

## Abstract

We monitored soil respiration (Rs), soil temperature (T) and volumetric water content (VWC%) over four years in one typical conventional and four alternative cropping systems to understand Rs in different cropping systems with their respective management practices and environmental conditions. The control was conventional double-cropping system (winter wheat and summer maize in one year - Con.W/M). Four alternative cropping systems were designed with optimum water and N management, i.e. optimized winter wheat and summer maize (Opt.W/M), three harvests every two years (first year, winter wheat and summer maize or soybean; second year, fallow then spring maize - W/M-M and W/S-M), and single spring maize per year (M). Our results show that Rs responded mainly to the seasonal variation in T but was also greatly affected by straw return, root growth and soil moisture changes under different cropping systems. The mean seasonal CO_2_ emissions in Con.W/M were 16.8 and 15.1 Mg CO_2_ ha^−1^ for summer maize and winter wheat, respectively, without straw return. They increased significantly by 26 and 35% in Opt.W/M, respectively, with straw return. Under the new alternative cropping systems with straw return, W/M-M showed similar Rs to Opt.W/M, but total CO_2_ emissions of W/S-M decreased sharply relative to Opt.W/M when soybean was planted to replace summer maize. Total CO_2_ emissions expressed as the complete rotation cycles of W/S-M, Con.W/M and M treatments were not significantly different. Seasonal CO_2_ emissions were significantly correlated with the sum of carbon inputs of straw return from the previous season and the aboveground biomass in the current season, which explained 60% of seasonal CO_2_ emissions. T and VWC% explained up to 65% of Rs using the exponential-power and double exponential models, and the impacts of tillage and straw return must therefore be considered for accurate modeling of Rs in this geographical region.

## Introduction

Soils provide a very large sink of carbon (C) in terrestrial ecosystems with C reserves of about 1500 Pg C (1 Pg = 10^15^ g) and make a major contribution to the global carbon equilibrium [1]. Slight changes in soil C might therefore lead to significant changes in the concentration of CO_2_ in the atmosphere. Soil respiration is the main terrestrial source of C return to the atmosphere with a flux reaching 98±12 Pg C in 2008 and increasing at a rate of 0.1 Pg C y^−1^ from 1989 [2]. Agricultural soils play a very important role in the global C cycle [3], [4] and account for 11% of global anthropogenic CO_2_ emissions [5]. It is therefore important to minimize soil respiration and retain more C sequestered in agricultural soils.

Soil respiration comprises mainly autotrophic respiration by plant roots and heterotrophic respiration of plant residues, root litter and exudates, and soil organic matter by soil microorganisms [6], [7]. Its magnitude is affected mainly by soil and climatic conditions [8] such as soil temperature and moisture [1], [9], [10], vegetation characteristics and management practices [11]–[14]. Soil respiration therefore shows high spatial and temporal variation [1]. Understanding this variation in different cropping systems in specific region will make a large contribution to the efficient management of C flow in agricultural ecosystems.

Soil respiration in cropland is greatly affected by tillage practices and straw management, with the greatest increase occurring immediately after tillage operations, and cumulative soil CO_2_ emissions can be lowered significantly by reducing the intensity of tillage [15], [16]. Daily CO_2_ fluxes can differ significantly at some sampling dates between conventional moldboard plow tillage and no tillage in continuous corn [17]. Soil CO_2_ emission can be enhanced in the short term after crop residues are returned to the field [15], [18] but this practice may build up the soil organic carbon (SOC) pools in the long term and may therefore be regarded as a more sustainable way of managing SOC compared to straw burning or other uses for straw [19]. Differences in fertilizer N rates had no significant effect on the CO_2_ exchange rates in the same crop rotation and CO_2_ fluxes did not differ with crop rotation under no till practices [16]. In addition, crop species and/or other management practices affect soil CO_2_ emission as a result of their influence on soil biological and biochemical properties [14], [20].

Soil temperature and moisture are two of the most important environmental factors controlling soil respiration [1], [21], [22]. Soil temperature is significantly positively correlated with soil respiration using linear [7], exponential [1], [7], improved Arrhenius [8], power and quadratic [9] and Q_10_ [10] models in different regions. Soil moisture is also a key factor controlling soil respiration, especially in arid or semiarid regions where it can be more important than temperature and become the dominant factor [11]. This shows that when one factor linking soil temperature and moisture is in a higher or lower range, the other might become a major factor controlling soil respiration [13], [23], [24]. The respiration rate will be limited when soil volumetric water content (VWC%) drops below a threshold of 15% [20]. Soil CO_2_ emission increased significantly with increasing temperature up to 40°C, with emissions reduced at the lowest and highest soil moisture contents [20]. Therefore, the single-factor models cannot describe soil CO_2_ emission well because they neglect the impacts of interactions between factors. The multiple polynomial models considering both soil temperature and moisture result in a much better description of CO_2_ (r^2^ = 0.70–0.78, *P*<0.0001) emissions than using temperature (r^2^ = 0.27–0.54, *P*<0.01) or moisture (r^2^ = 0.29–0.45, *P*<0.01) alone [20].

China has broad climatic regimes and the different ecosystems depend on regional climatic conditions [25]. The North China Plain (NCP) is a major agricultural region. The soil type is Fluvo-aquic soil and the climate is sub-humid temperate monsoon with abundant solar radiation but with cold and dry conditions in winter and spring and warm and wet weather in summer. Evapo-transpiration is intense and the spring drought is an important feature. Winter wheat-summer maize is the typical double cropping system and current farming practice involves application of 300 kg N ha^−1^ yr^−1^ for winter wheat and 250 kg N ha^−1^ yr^−1^ for summer maize with a ratio of basal to topdressing applications of 1∶1 and 1∶1.5, respectively [26], [27]. The soil is rotary tilled to 20 cm depth after maize straw removal for sowing winter wheat, and maize is sown directly after removing the wheat straw. Generally, wheat is irrigated three to four times and maize once or twice depending on precipitation. The amount of irrigation water ranges from 60 to 100 mm on each occasion [26]. About 30–60% of N input could be saved without sacrificing yields while significantly reducing environmental risk by adopting optimum N management in the winter wheat-summer maize system as shown by our earlier study [28]. However, over-exploitation of groundwater has become the main factor restricting sustainable agricultural development [29]. There is therefore concern to explore new alternative cropping systems for sustainable use of groundwater and optimum N fertilization to reduce pollution. Winter wheat–summer maize–spring maize with three harvests over two years and a single spring maize system have shown great potential to reduce water use and N use and can achieve balanced use of groundwater [26], and this cropping system may serve as a new alternative system for efficient resource use and sustainable development. However, it is still unclear how these changes will affect soil respiration in the study region.

Low frequency of measurement, lack of data at some growth stages, and failure to consider the interactive effects of soil moisture and temperature on soil respiration may lead to failure to describe the characteristics of soil respiration in this region [18], [30], [31]. There are indications that the correlation between soil respiration and soil temperature to 5 cm depth is 0.51 but the study that produced this result involved measurement only 21 times over one year [30]. Meng et al. [31] found that soil respiration had a higher correlation with soil temperature to 5 cm depth using the exponential model through weekly measurements of soil respiration under the typical double-cropping system over a whole year. Soil temperature at 5 cm depth explained 63–74% of soil respiration using the exponential model except during the winter, and the application of crop residues had significant positive impacts on soil respiration [18]. The management of N and water, crop residues and tillage practices will change significantly after conversion to new alternative cropping systems [26], an effect closely related to soil respiration. However, no quantitative information is yet available regarding soil respiration in new alternative cropping systems in this region.

In the present study we have compared soil respiration characteristics in different cropping systems with their respective management practices and environmental variables and we explore the factors affecting these differences. We have also analyzed the effects of straw return on variation in seasonal CO_2_ emissions on the North China Plain.

## Materials and Methods

### Site description

**Figure 1:**
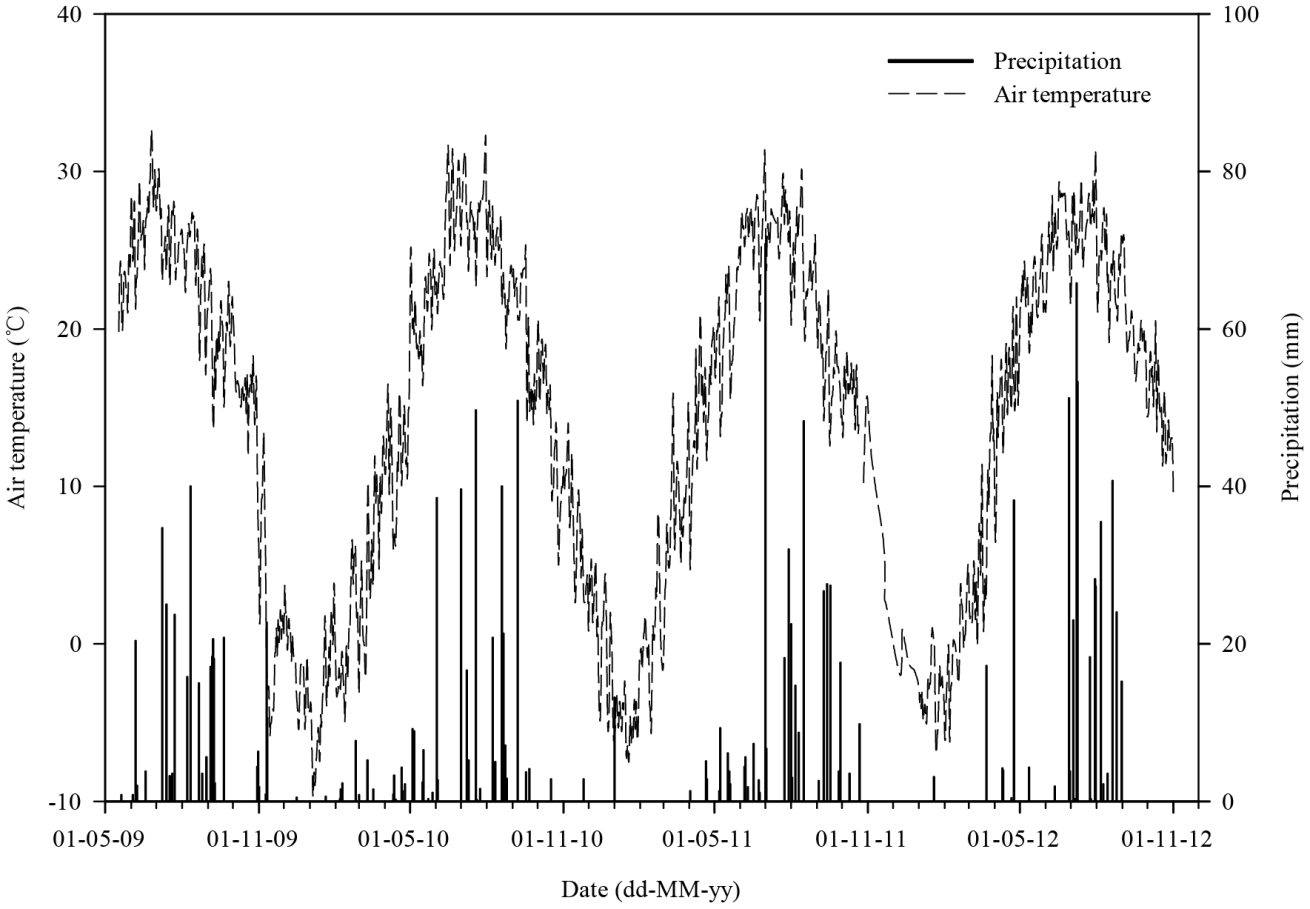
Daily mean air temperature (°C) and precipitation (mm) during the field experiment.

A long-term field experiment was set up in October 2007 at Quzhou experimental station (36.87°N, 115.02°E) of China Agricultural University in Hebei province. The site is a sub-humid temperate monsoon area at an altitude of 40 m. The annual mean temperature is 13.2°C. Annual mean precipitation was 494 mm from 1980 to 2010 with a range of 213–840 mm, and 68% of precipitation falls from June to September [26]. The typical double-cropping system is a winter wheat and summer maize rotation which accounts for >80% of agricultural fields in Quzhou county. The soil type is Fluvo-aquic soil and the bulk density of the top 30 cm of the soil profile is 1.37 g cm^−3^, soil pH is 7.72 (1∶2.5, soil∶water), SOC content 7.31 g kg^−1^, total N 0.7 g kg^−1^, Olsen-P 4.8 mg kg^−1^ and available K 72.7 mg kg^−1^. Fig. 1 shows the daily mean air temperatures and precipitation during the measurement period (also see Table S1).

### Field experiment treatments and management

A completely randomized design was employed with five treatments and four replicates. Each plot is 1800 m^2^ (30×60 m). The control is conventional winter wheat and summer maize based on local farming practice (Con.W/M). Four new alternative cropping systems were designed with high-yielding varieties (using optimum planting density and crop management) and optimum water and N fertilizer management compared with conventional practice. They are: optimized two harvests in one year (winter wheat and summer maize - Opt.W/M), three harvests within two years (first year, winter wheat and summer maize or winter wheat and summer soybean; second year fallow then spring maize - W/M-M and W/S-M) and single spring maize per year (M).

Nitrogen input and irrigation for Con.W/M were described in the Introduction above. The basal fertilizer for wheat was surface broadcast before rotary tillage to 20 cm depth after removal of maize straw from the soil and topdressing was broadcast at shooting for wheat followed by irrigation, with both fertilizer applications at 150 kg N ha^−1^ in the form of urea. The basal application for summer maize comprised 45 kg N ha^−1^ applied to the soil as 15-15-15 compound fertilizer with a seed drill after removing the wheat straw from the soil, and 55 kg N ha^−1^ surface broadcast as urea followed by irrigation and the topdressing 150 kg N ha^−1^ was applied at the ten-leaf stage of summer maize in the form of urea. In the other systems optimized N management was devised according to the N target values minus the soil nitrate-N content in the root zone before side-dressing as described by Cui et al. [27]. For summer maize 45 kg N ha^−1^ was applied as a basal dressing in the same way as for Con.W/M and 80 and 60 kg N ha^−1^ were side-dressed using a soil cover of 0–5 cm after band application at the six- and ten-leaf stages of summer maize, respectively. No other N fertilizer except 45 kg N ha^−1^ was applied as a basal application for soybean as for Con.W/M. Irrigation times and rates were determined by testing the soil water content before the critical growing seasons as described by Meng et al. [26]. The details of nitrogen input and irrigation rate over the whole study are shown in Table 1. Wheat straw was mulched after chopping into 5–10 cm pieces and summer maize or soybean was sown directly. Summer and spring maize and soybean residues were also chopped into 5–10 cm pieces and mechanically ploughed into the top 30 cm of the soil after maize and soybean were harvested and then winter wheat was sown if there was no fallow the following season. The soil was rotavated to 20 cm depth before sowing spring maize.

**Table 1:** Nitrogen fertilizer rates and irrigation rates throughout the study period.

Year	N application rate (kg N ha^−1^)					Irrigation rate (mm)				
	Con.W/M^1^	Opt.W/M	W/M-M	W/S-M	M	Con.W/M	Opt.W/M	W/M-M	W/S-M	M
2009	W^2^ 300	W 263	F -3	F -	F -	W 250	W 215	F -	F -	F -
	M_1_ 250	M_1_ 185	M_2_ 135	M_2_ 210	M_2_ 95	M_1_ 60	M_1_ 60	M_2_ 125	M_2_ 135	M_2_ 125
2010	W 300	W 100	W 140	W 140	F -	W 180	W 120	W 120	W 120	F -
	M_1_ 250	M_1_ 185	M_1_ 185	S 45	M_2_ 150	M_1_ 60	M_1_ 60	M_1_ 60	S 60	M_2_ 110
2011	W 300	W 139	F -	F -	F -	W 240	W 275	F -	F -	F -
	M_1_ 250	M_1_ 185	M_2_ 162	M_2_ 178	M_2_ 150	M_1_ 70	M_1_ 70	M_2_ 60	M_2_ 60	M_2_ 60
2012	W 300	W 140	W 162	W 158	F -	W 180	W 160	W 160	W 170	F -
	M_1_ 250	M_1_ 185	M_1_ 185	S 45	M_2_ 266	M_1_ 90	M_1_ 90	M_1_ 90	S 90	M_2_ 120
Total	2200	1382	969	776	661	1130	1050	615	635	415

1 Con.W/M, Opt.W/M, W/M-M, W/S-M and M represent conventional and optimized winter wheat–summer maize, winter wheat–summer maize–spring maize, winter wheat–summer soybean–spring maize and spring maize treatment, respectively.

2 W, M_1_, M_2_, S and F represent winter wheat, summer maize, spring maize, summer soybean and fallow.

3 Denotes no data in the fallow season.

**Figure 2:**
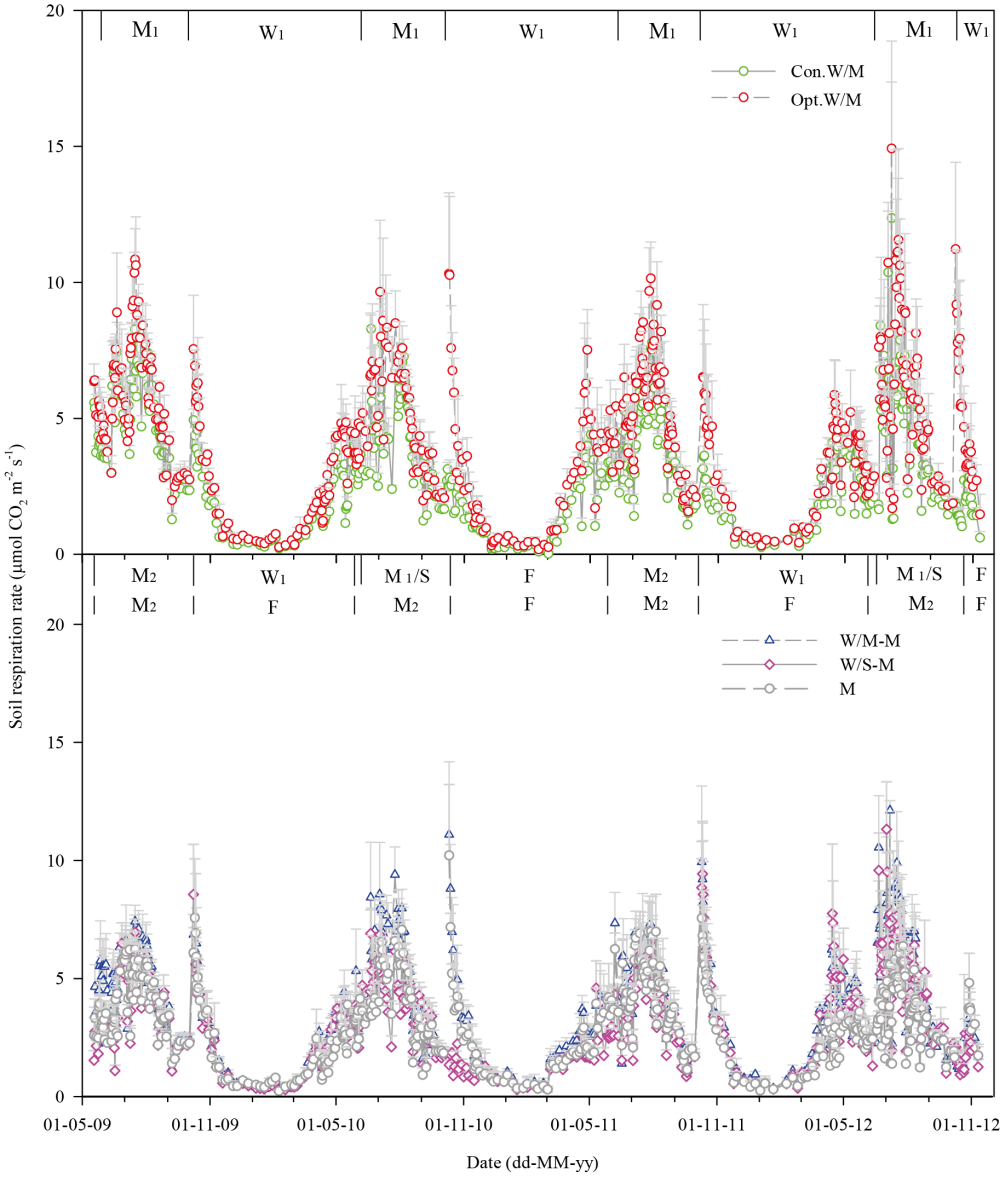
CO_2_ emissions of different cropping systems. Con.W/M, Opt.W/M, W/M-M, W/S-M and M represent conventional winter wheat–summer maize in one year, optimized winter wheat–summer maize in one year, winter wheat–summer maize (or summer soybean) –spring maize three harvests in two years and single spring maize system in one year; W, M_1_, M_2_, S and F represent winter wheat, summer maize, spring maize, soybean and fallow.

We measured soil respiration in each plot of the experiment from May 2009 to October 2012. Crops present in the different treatments during gas measurement are shown in Fig. 2.

### Soil respiration measurement

Soil respiration was representatively determined in every plot using an automatic soil CO_2_ flux system (LI-COR LI-8100, Lincoln, NE). Measurements were carried out daily for 10 days after fertilization events and 3–5 days after irrigation or precipitation events (>10 mm) depending on the size of gas fluxes; for the remaining periods emissions were measured twice per week and once a week when the soil was frozen. Two bases were used in each plot, one on a row and the other in the middle of the row during the maize and soybean seasons. Each base was a PVC tube with an inner diameter of 20 cm and a height of 13 cm inserted 9 cm into the soil for measurement and was removed only before sowing. Soil respiration was measured directly by LI-8100 in units of µmol CO_2_ m^−2^ s^−1^ in the field between 08:30 and 11:00 am. Soil respiration is presented as the mean values of four replicated measurements on four different plots. The seasonal amounts of CO_2_ emissions were sequentially linearly determined from the emissions between every two adjacent intervals of the measurements.

### Auxiliary measurements

Soil temperature to 5 cm depth was measured directly by Li-8100 through a temperature sensing probe during the measurement time. Soil moisture at 0–5 cm is expressed as volumetric water content (VWC%) and was measured directly by Li-8100 through an ECH_2_O type of EC-10 soil water sensing probe (Decagon Devices, Inc, Pullman, WA). We also measured the top 20 cm depth SOC content in each plot of this field experiment after summer maize harvest in 2011 using the method described by Huang et al. [19]. The daily mean air temperatures and precipitation data during the field experiment were obtained from an automatic weather station located 50 m from our experimental site as shown in Fig. 1. Soil respiration and environmental variable data from the present study are presented in the Supplementary Data (Table S1).

### Correlations between soil respiration and soil temperature and moisture

The compound factor models of soil respiration with soil temperature and moisture (equations 1–4) were employed as follows:
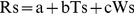
(1)

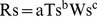
(2)

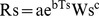
(3)

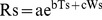
(4)


We established the four compound factor models above among soil respiration (Rs), soil temperature (Ts) and VWC(%) (Ws) using the measured fluxes from May 2009 to May 2012, and compared MAE (the mean absolute error), ME (model efficiency, the ratio of difference in measured and predicted flux in total variation in measured flux, expressed as significant correlation coefficient from −1 to 1), d (the percentage of mean square error and potential error, expressed as significant correlation from 0 to 1) [32], [33], RMSE (root mean square error, reflecting the degree of dispersion of one variable), MSE_s_ (systematic error) and MSE_u_ (random error) [34] among the models. We comprehensively evaluated the model performances by the sizes of MAE, ME, d, RMSE, MSE_s_ and MSE_u_, and the value of MSE_s_/(MSE_u_+MSE_u_). In general, MSE_u_ is close to RMSE in a well fitting model.

### Statistical analysis

The primary data were processed using Microsoft Excel 2003 spreadsheets. Total CO_2_ emissions in the different treatments were tested by analysis of variance and mean values were compared using SAS statistical software (Version 9.2; SAS Institute, Inc., Cary, NC) to calculate least significant difference (LSD) at the 5% level. Compound factor regression analysis among soil respiration, T and VWC% were performed using Sigmaplot 12.0 (Systat Software Inc., Erkrath, Germany).

## Results

### Characteristics of soil respiration in the different cropping systems

**Figure 3:**
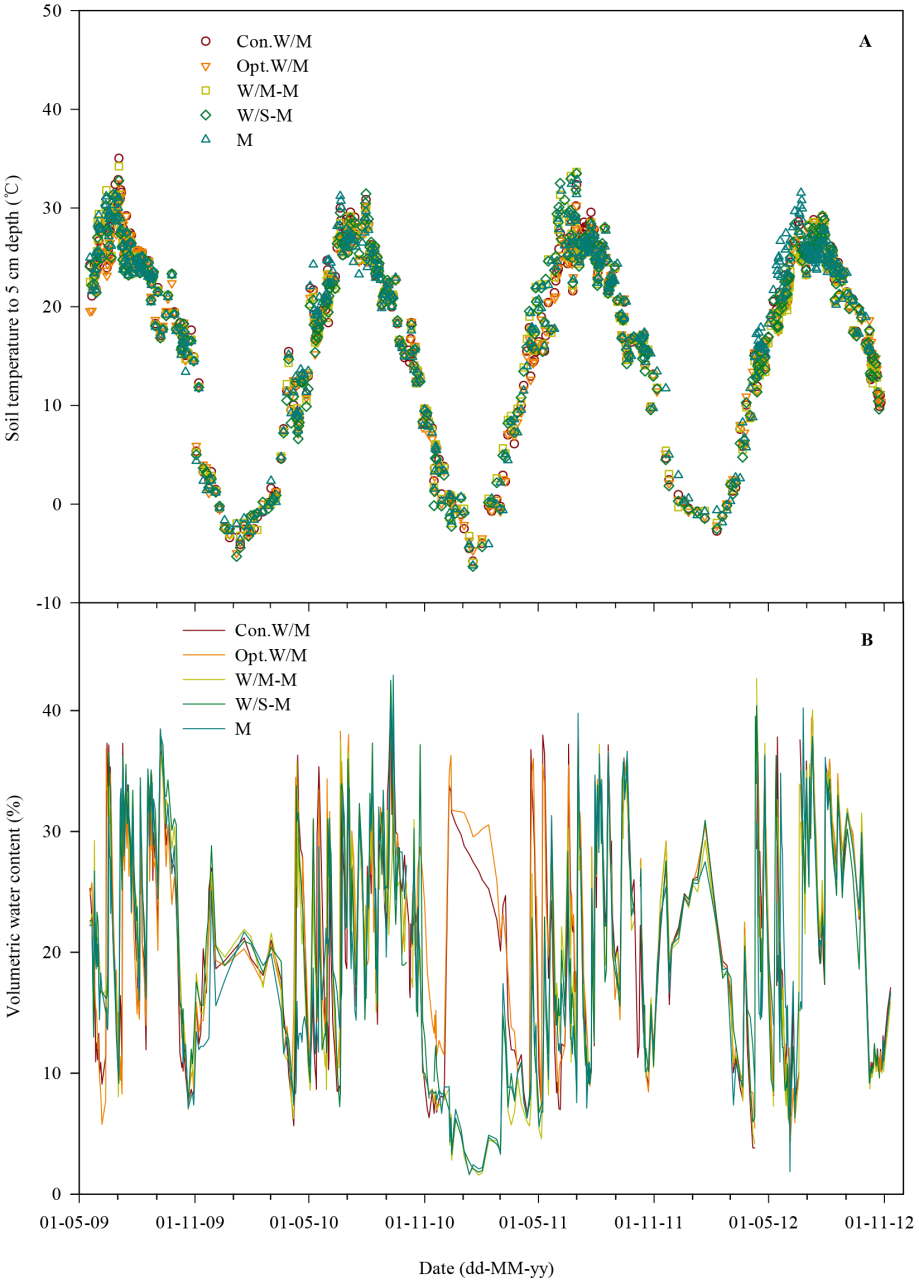
Dynamics of (A) soil temperature and (B) soil VWC% to 5 cm depth.

Over a complete rotation cycle soil respiration gradually increased from March, reached a maximum in July and gradually decreased from August to November, and then remained at the lowest values during winter, in a pattern similar to soil temperature (Figs. 2 and 3A). The mean soil respiration values were 3.35, 4.55, 4.03, 3.35 and 3.25 µmol CO_2_ m^−2^ s^−1^ for Con.W/M, Opt.W/M, W/M-M, W/S-M and M throughout the study period, with ranges of 0.02–12.4, 0.26–14.9, 0.31–12.1, 0.34–11.3 and 0.30–11.2 µmol CO_2_ m^−2^ s^−1^, respectively. Three peaks per year occurred in the typical double-cropping system, at the shooting stage of winter wheat, six-leaf of summer maize and the period after winter wheat sowing, the first two peaks caused by rapid crop growth and the last by the return of summer maize straw combined with soil tillage. Soil respiration of Opt.W/M was higher than of Con.W/M at the six-leaf stage of summer maize in the middle of July and the period after winter wheat sowing. The maximum peaks of soil respiration in Con.W/M were 8.2, 7.7, 7.8, 12.4 and 4.9, 2.8, 3.6, 2.9 µmol CO_2_ m^−2^ s^−1^ during these two periods for four growing seasons, respectively, and they increased to 10.8, 9.6, 10.1, 14.9 and 7.6, 10.3, 6.5, 11.2 µmol CO_2_ m^−2^ s^−1^ in Opt.W/M during the corresponding periods. Under the new alternative cropping systems one peak disappeared in the fallow season (season with no winter wheat planted). The highest value of soil respiration was around 7.0 µmol CO_2_ m^−2^ s^−1^ in the spring maize season under the new alternative cropping systems, but it increased to more than 10.0 µmol CO_2_ m^−2^ s^−1^ for summer maize in Opt.W/M at the corresponding time (Fig. 2). Soil respiration was very low even after summer soybean stover return to the field in W/S-M in mid-November 2010 when the soil temperature in the top 5 cm ranged from −2.3 to +4.7°C within a month of soil tillage. A similar phenomenon occurred at the end of October 2012 due to the late spring maize and summer soybean harvests and the soil was tilled when soil temperature to 5 cm depth was around 10°C, and the peaks of W/M-M, W/S-M and M were only one third of the values of those at the corresponding times in other years. In addition, soil respiration showed large between-year change, so that peaks of soil respiration occurred after irrigation at shooting of winter wheat in other years, but not in winter wheat in 2010.

### Total CO_2_ emissions in each cropping season and each rotation cycle

Total CO_2_ emissions in each cropping season and each rotation cycle were system dependent (Table 2). The mean seasonal total CO_2_ emissions of Con.W/M were 16.8 and 15.1 Mg CO_2_ ha^−1^ for summer maize and winter wheat, respectively. They increased significantly by 26 and 35% in Opt.W/M in the corresponding season. Under the new alternative cropping systems W/M-M showed similar results to Opt.W/M, and the seasonal total CO_2_ emission of W/M-M was significantly higher than the corresponding season of Con.W/M except spring maize in 2009. However, W/S-M showed no significant difference from Con.W/M in each cropping season and the total CO_2_ emissions in the fallow season and spring maize of W/S-M were clearly affected by summer soybean planting. Total CO_2_ emission of M in each cropping season also showed no clear difference from Con.W/M except spring maize in 2011. In order to compare the impacts of cropping systems on CO_2_ emissions of each rotation cycle we calculated the total CO_2_ emissions during the period 2011–2012, which included two rotation cycles of Con.W/M, Opt.W/M, and M and a completely rotation cycle of W/M-M and W/S-M. Total CO_2_ emission of Con.W/M was 61.9 Mg CO_2_ ha^−1^, and increased significantly by 37 and 29% in Opt.W/M and W/M-M treatment, respectively. The total CO_2_ emission of W/M-M was not significantly different from Opt.W/M when there was only one season of winter wheat in two years, but total CO_2_ emission of W/S-M decreased sharply in contrast to Opt.W/M when summer soybean was planted to replace summer maize of W/M-M because soil respiration was reduced significantly in the following fallow and spring maize seasons after the low biomass of soybean straw was returned to the soil. Total CO_2_ emissions expressed as one complete rotation cycle of W/S-M, Con.W/M and M treatments were not significantly different (Table 2).

**Table 2:** Total CO_2_ emissions in each cropping season and each rotation cycle (Mg CO_2_ ha^−1^).

Year	Con.W/M		Opt.W/M	W/M-M		W/S-M		M	
	Crop	CO_2_	CO_2_	Crop	CO_2_	Crop	CO_2_	Crop	CO_2_
2009	M_1_	19.1±0.8bc^1^	22.0±1.5a	M_2_	21.4±1.9ab	M_2_	17.5±1.3c	M_2_	17.4±1.4c
2010	W	15.5±1.2bc	17.2±0.6ab	W	17.8±1.2a	W	17.0±0.9abc	F	14.9±0.5c
	M_1_	16.0±0.9b	21.4±1.7a	M_1_	20.6±1.8a	S	15.7±0.9b	M_2_	18.6±2.5ab
2011	W	13.4±0.7bc	22.4±0.4a	F	19.6±0.4a	F	11.1±1.6c	F	15.9±1.6b
	M_1_	15.5±1.4b	20.4±1.9a	M_2_	19.6±2.1a	M_2_	16.0±1.1b	M_2_	18.7±1.2a
2012	W	16.5±0.9bc	21.7±3.5a	W	21.3±2.4a	W	19.3±1.6ab	F	15.0±1.8c
	M_1_	16.4±0.6c	20.4±1.9a	M_1_	19.4±1.4ab	S	17.0±2.0bc	M_2_	18.0±0.7abc
2009-2012 Mean	M_1_	16.8	21.1	M_1_	20.0	S	16.4	-	
	W	15.1	20.4	W	19.6	W	18.2	-	
	-	-	-	M_2_	20.5	M_2_	16.8	M_2_	18.2
	-	-	-	F	19.6	F	11.1	F	15.3
2011-2012	2 W-M_1_ 2	61.9±1.3b	84.9±8.2a	F-M_2_-W-M_1_	79.8±5.6a	F-M_2_-W-S	64.1±2.7b	2 F-M_2_	67.1±2.0b

1 The same letter in the same line denotes no significant difference in different cropping systems by LSD at *P*<0.05.

2 2 W-M_1_, F-M_2_-W-M_1_ (or S) and 2 F-M_2_ represent two winter wheat-summer maize rotation cycles, fallow-spring maize-winter wheat-summer maize (or summer soybean) rotation cycle and two fallow-spring maize rotation cycles.

### Soil respiration as affected by C input in each growth season

**Figure 4:**
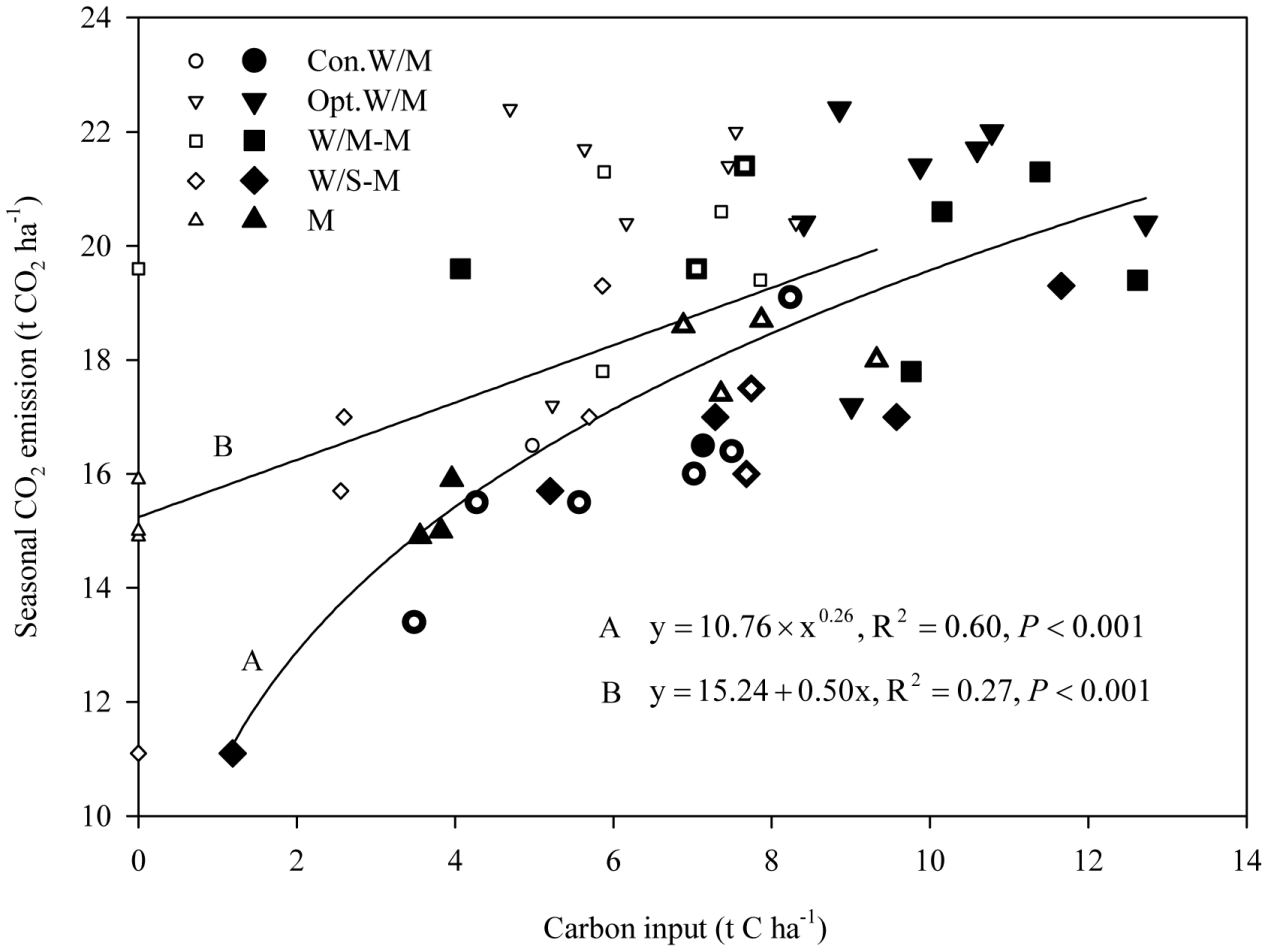
Correlation between seasonal CO_2_ emission and carbon input. Carbon input was calculated from current-season aboveground biomass only (A); and calculated from straw return of the previous season and the aboveground biomass in the current season (B); the abbreviations of the treatment are shown in the footnotes in Fig. 2.

The measured soil respiration rates in this study consisted mainly of autotrophic respiration by crop roots in the current season, heterotrophic respiration of root litter and exudates in the current season, and heterotrophic respiration of crop straw return to the soil from the previous season and soil organic matter. As Fig. 2 and Table 2 show, the characteristics and total seasonal cumulative CO_2_ emissions were greatly affected by straw return and crop growth status. To further explain soil respiration driven by C input in each growing season, we analyzed the correlation of seasonal cumulative CO_2_ emissions with: (1) current-season aboveground biomass only; (2) the sum of C input of straw return from the previous season and the aboveground biomass in the current season. The relationship is improved significantly by inclusion of straw inputs (Fig. 4, equation A) compared to current-season aboveground biomass only (Fig. 4, equation B). Carbon input of straw return from the previous season and the aboveground biomass in the current season explains up to 60% of seasonal cumulative CO_2_ emissions, much higher than that of 27% with current-season aboveground biomass only, which demonstrates that straw C inputs from the previous season can significantly affect soil respiration.

### Correlation between soil respiration and soil temperature and VWC% to 5 cm depth

**Figure 5:**
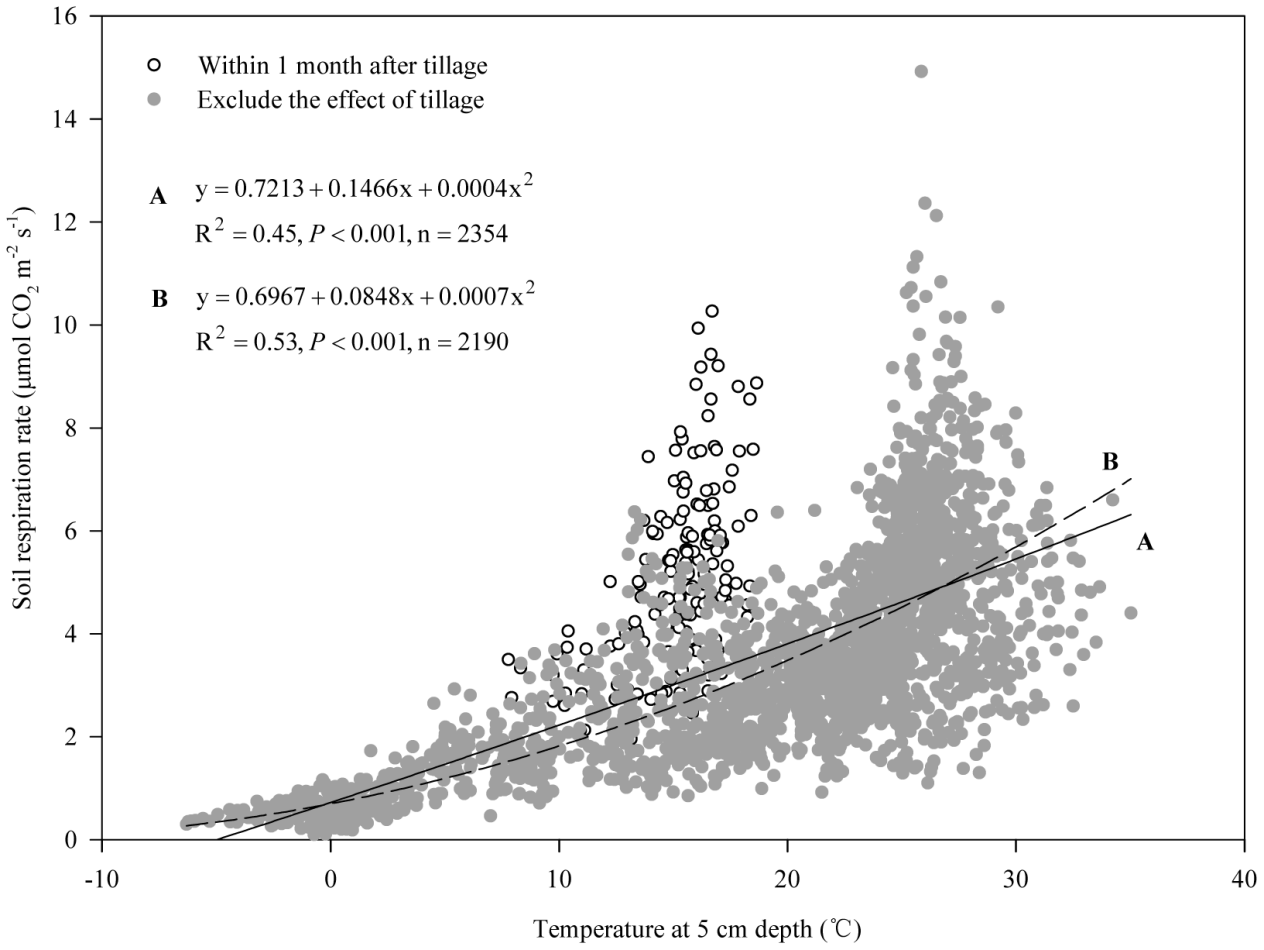
Impacts of soil tillage combined with straw return on soil respiration. Correlation between soil respiration and soil temperature at 5(equation A) and the correlation between soil respiration and soil temperature at 5 cm depth excluding the data within one month of tillage (equation B).

Large changes in soil respiration followed the variation in temperature over a complete year (Figs. 2 and 3A). Soil temperature explained 45% of soil respiration using the quadratic model (Fig. 5, equation A). In addition, soil moisture exerted some impacts on soil respiration under our climatic conditions such as inhibition within a short period after irrigation at shooting and grain filling stages of winter wheat and then a sharp increase which was derived from the effects of drying and wetting cycles. Soil respiration showed significant correlations with soil VWC% using the linear (R_s_ = 2.7535+0.0447V, R^2^ = 0.04, n = 2282) and power (R_s_ = 1.7708V^0.2488^, R^2^ = 0.06, n = 2282) models at *P*<0.001. However, soil VWC% explained only 4–6% of soil respiration. We further examined the combined effects of soil temperature and VWC% using four compound models, namely the linear, power, exponential-power and double exponential models (Table 3). The results indicate that the R^2^ values combining temperature and VWC% are significantly higher than using the quadratic model only considering soil temperature. Soil temperature and VWC% explained up to 65% of soil respiration using the exponential-power and double exponential models.

**Table 3:** Correlation between soil respiration and soil temperature and VWC(%) to 5 cm depth.

Model	Fitting equation	n	R^2^	MAE	ME	RMSE	MSE_s_	MSE_u_	d
Linear	R_s_ 1 = 0.7712+0.1581T – 0.0030V	1905	0.47*^2^	1.11	0.48	1.48	1.28	1.03	0.80
Power (T>0)	R_s_ = 0.6291T^0.5929^V^-0.0096^	1811	0.56*	1.17	0.36	1.57	2.13	0.59	0.70
Exponential-power	R_s_ = 0.9347e^0.069T^V^-0.0464^	1905	0.65*	1.14	0.38	1.61	1.41	1.67	0.80
Double exponential	R_s_ = 0.8924e^0.0693T-0.0045V^	1905	0.65*	1.20	0.31	1.69	1.36	1.81	0.78

1 R_s_, T and V represent soil respiration, soil temperature and VWC% to 5 cm depth, respectively.

2 * represents highly significant correlation at *P*<0.001.

The exponential-power and double exponential models (Table 3) gave significant improvements compared to the linear and power models. We again compared MAE, ME and d, RMSE, MSE_s_ and MSE_u_ among the four models and comprehensively evaluated the model performances by the sizes of these indicators and the value of MSE_s_/(MSE_u_+MSE_u_). The exponential-power model was much better for description of soil respiration in response to soil temperature and VWC% (both in the top 5 cm) in our study because it had lower MAE, RMSE, MSE_u_, and higher ME than the double exponential model and the values of MSE_s_/(MSE_s_+MSE_u_) were similar using both models.

## Discussion

**Figure 6:**
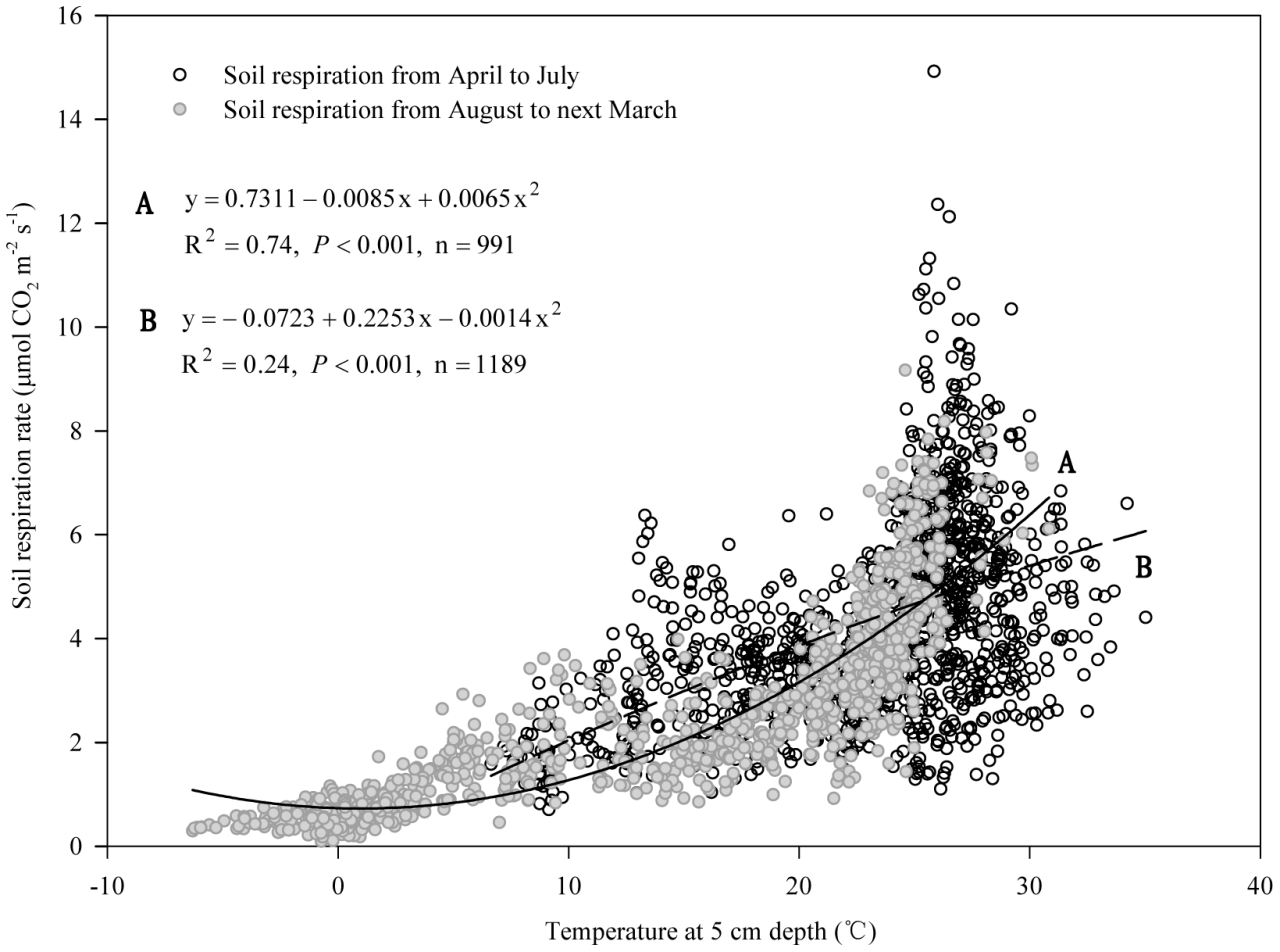
Correlation between soil respiration and soil temperature at 5 cm depth. Equations A and B represent the correlations between soil respiration and soil temperature at 5

Soil respiration in croplands is affected mainly by soil properties, cropping system (which is related to crop species), tillage and straw management, water and nutrient management, and environmental variables (soil temperature, moisture etc.) [1], [6], [20], [35]. There is temporal variation within the same cropping system and spatial variation among different cropping systems [16], [17], [31]. Changes in soil respiration in our sub-humid temperate monsoon region are largely affected by the seasonal variation in temperature, which is in line with most previous reports [30], [31], [36]. However, soil respiration responded little to soil temperature as shown in Fig. 5, equation A using the quadratic model because some data points did not fit the model with the impacts of soil tillage before the wheat crop was sown. The R^2^ value improved by 18%, and up to 53% when the data within one month after tilling were excluded (Fig. 5, equation B). Moreover, we found that soil respiration tended to follow the variation in temperature from August to the following March when the data after tillage were excluded (Figs. 2 and 3A). Soil temperature explained 74% of soil respiration when only the data from August to March were included (Fig. 6, equation A). Therefore, the impacts of tillage must be considered for modeling soil respiration on the NCP.

The short decline in soil respiration after irrigation might be attributable to blocked diffusion of CO_2_ with high moisture and limited oxygen concentrations in the soil matrix [37], and the flushes afterwards may be due to the stimulation of decomposition of plant residues [21], root litter and exudates or autotrophic respiration of rapid root growth, which taken together induced the effects of drying and wetting cycles. Soil respiration would be limited when soil moisture was too high or too low and the maximum range is usually close to field water holding capacity [38]. The disappearance of respiration flushes was due to the low soil temperatures within a week after irrigation at the shooting stage of wheat in 2010 relative to other years (6–10°C in 2010 vs 12–21°C in 2011 and 12–19°C in 2012) (Fig. 3A). Soil moisture was not the key driving factor over the whole study period but did affect soil respiration slightly at particular stages and therefore only explained a very small proportion of the variation in soil respiration in our study area.

Numerous studies have reported that soil respiration is significantly affected by tillage practices combined with straw management [14]–[16]. Total soil respiration was significantly higher in Opt.W/M than Con.W/M as the latter soil was rotary tilled to 20 cm depth after maize straw removal and Opt.W/M was ploughed into the top 30 cm of the soil after maize straw return to the soil, soil respiration increased sharply after soil disturbance by tillage operations possibly because increased soil aeration accelerated the decomposition rate of crop residues which was associated with higher microbial activity [14], [15], [39]. However, the impacts of maize straw return and tillage were lowered by delaying tillage until the soil temperature to a depth of 5 cm reached 10°C or lower.

Although seasonal cumulative CO_2_ emission in Opt.W/M and W/M-M increased significantly relative to Con.W/M as a result of straw return [14], [15], this practice also increases the SOC content over the long term [16], [17], [40]. The SOC content in the top 20 cm of the soil profile in straw return treatments increased by 3.9–16.5% relative to the straw removal treatments in winter wheat–summer maize double-cropping systems on the NCP, with a mean increase in rate of 0.04 to 1.44 t C ha^−1^ y^−1^ over a six-year period as shown by Huang et al. [19]. We also measured SOC to a depth of 20 cm in the present field experiment after summer maize harvest in 2011 and all values increased to 8.07, 8.71, 7.93 and 7.52 g kg^−1^ in Con.W/M, Opt.W/M, W/M-M and W/S-M, respectively, with the sole exception of a slight decrease to 7.18 g kg^−1^ in M (from 7.31 g kg^−1^ at the start of the field experiment in 2007). Although there was no crop straw return, Con.W/M also showed a clear increment relative to the initial value in line with Huang et al. [19], and this may have been due to the large amounts of crop roots and rhizo-deposited carbon. Con.W/M showed a greater increase in SOC than W/M-M, W/S-M and M, possibly due to the lower intensity of tillage in Con.W/M than in W/M-M, W/S-M and M. Our results show that soil respiration responded mainly to the seasonal variation in soil temperature but was also greatly affected by straw return, root growth and soil moisture changes under the different cropping systems.

## Supporting Information

Table S1 Measured soil respiration fluxes, soil temperature and soil volumetric water content to 5 cm depth, daily mean air temperature and precipitation in the field experiments from 18^th^ May 2009 to 11^th^ November 2012.
(XLS)
