# Correction: Comparison of Soil Respiration in Typical Conventional and New Alternative Cereal Cropping Systems on the North China Plain

**DOI:** 10.1371/annotation/e7f51456-9b9e-4766-966f-a252bc2ca4f1

**Published:** 2013-12-03

**Authors:** Bing Gao, Xiaotang Ju, Fang Su, Fengbin Gao, Qingsen Cao, Oene Oenema, Peter Christie, Xinping Chen, Fusuo Zhang

There were multiple errors in the Funding Statement. The Funding Statement should read: "This work was funded by the ‘973’ Project (2012CB417105, 2009CB118606), the National Natural Science Foundation of China (41230856, 31172033), the Special Fund for the Agricultural Profession (201103039), China Postdoctoral Science Foundation (2013M 530778), and the Innovation Group Grant of the National Natural Science Foundation of China (31121062). The funders had no role in study design, data collection and analysis, decision to publish, or preparation of the manuscript." 

